# Modulating the avian cecal microbiome: probiotics in the prevention of *Histomonas meleagridis* in poultry

**DOI:** 10.3389/fvets.2026.1901540

**Published:** 2026-08-12

**Authors:** Suqin Wang, Aftab Shaukat, Meihua Zhou, Jingwen Tang, Fangyu Liu, Congqiang Luo

**Affiliations:** 1 College of Life and Environmental Sciences, Hunan Provincial Key Laboratory of Zoology, Changde Research Center for Agricultural Biomacromolecule, Hunan University of Arts and Science, Changde, Hunan, China; 2 College of Synthetic Biology Industry, Hunan University of Arts and Science, Changde, Hunan, China

**Keywords:** antimicrobial alternatives, *Histomonas meleagridis*, histomoniasis, immunomodulation, microbial dysbiosis, probiotics

## Abstract

Histomoniasis is a serious infectious enteric disease that mainly affects gallinaceous poultry in all parts of the world. The protozoan parasite, *Histomonas meleagridis* is the causative agent. Once regulated by synthetic chemical additives, the disease has resurged globally since the banning of effective yet probably toxic pharmacological prophylactic agents. The pathogenesis of *Histomonas meleagridis* has a complicated interaction with the avian cecal microbiome, which is discussed in this review. Infection leads to active induction of deep dysbiosis by the parasite that drastically decreases the number of beneficial bacteria and increases growth of opportunistic pathogens. The scientifically feasible option of mitigating diseases by modulating the cecal ecosystem with targeted probiotic administration has come up. This review explains the biological processes of the parasite, the homeostatic role of the microbiome, and the high standards of selection of poultry probiotics. Crucially, while most current evidence remains indirect due to a lack of targeted *in vivo* models, this review uniquely explains how probiotics utilize competitive exclusion and immunomodulatory mechanisms to reverse parasitic colonization. By comparing these microbial interventions to existing avian prophylaxis, this manuscript highlights the practical significance of precision microbiome modulation in commercial flocks and sets the stage for future targeted experimental research.

## 1 Introduction

The poultry sector worldwide is one of the pillars of contemporary agricultural production and a critical source of quality protein for the human body (1). It is important to keep the flock in optimal health to ensure sustainable yields of production and protection of food security (2). Nevertheless, the commercial poultry industry is under constant risk from numerous infectious factors which can easily undermine the welfare of the animals and their economic sustainability (3). One of these major threats is the histomoniasis disease that is popularly referred to as blackhead disease by the agricultural community (4). It is a serious disease that impacts mostly on turkeys and chickens, and is caused by the protozoan parasite, *Histomonas meleagridis* (5). Infection easily results in outbreaks in susceptible turkey flocks with mortality rates that can be up to 100 % without intervention (6). Chickens have a lower mortality rate but may be asymptomatic carriers, and their growth performance and egg production drop (7). In the past, agricultural sector dealt with the outbreak of histomoniasis by adding synthetic chemical substances into either feed or water (5). Arsenical derivatives and nitroimidazole compounds have been used for decades and have been found to offer excellent prophylactic and therapeutic effects against the parasite (8). These pharmacological interventions helped farmers to avoid disastrous losses of the flocks (9). Consequently, mounting scientific evidence on the possible toxicity and carcinogenicity of these synthetic drugs, and this cast serious health concerns on the population (10). As a result, regulatory bodies globally implemented stringent legislative measures to safeguard consumers (11). In the late nineteen nineties to two thousand three, the European Union officially prohibited the use of any preventive and curative drugs on *Histomonas meleagridis* in food-producing animals (5). In the United States, in two thousand fifteen, the Food and Drug Administration also did so by revoking the approval of the last prophylactic arsenical compound on the market, nitarsone (12).

The sudden withdrawal of these useful chemical treatments created a significant therapeutic void in commercial poultry management (13). In the absence of a feasible pharmaceutical countermeasure, there was a rapid and massive re-emergence of histomoniasis outbreaks in farmers (5). The traditional and free-range poultry business was devastated by huge losses of birds; recent epidemiological data indicates that histomoniasis outbreaks can cause up to 100% mortality in turkey flocks and significant drop in egg production in chickens, leading to heavy global economic burdens (6, 14). This crisis underscored an urgent need to develop novel and safe prophylactic measures that satisfy modern regulatory requirements and align with consumer demands of antibiotic-free meat (15). Over the past years, scientists have begun to focus more on exploring the underlying biological mechanisms of enteric pathogen-host gastrointestinal ecosystem interactions (16). A very high density and sophisticated community of microorganisms resides in the avian cecum and is referred to as the cecal microbiome (17, 18). This complex microbial community has a primary role of ensuring proper homeostasis of the intestines, optimum digestion of nutrients, and training the host immune system (19). Moreover, the healthy and even-handed cecal microbiome gives a very important protective mechanism regarding colonization and growth by invading pathogens through competition (20, 21). Recent scientific studies have conclusively proved that in case of infection, *Histomonas meleagridis* interferes with this delicate balance of microbes (22). This interruption results in a severe condition of dysbiosis with a dramatic drop in the number of beneficial bacteria and their subsequent overpopulation with opportunistic pathogens such as *Escherichia coli* (23).

The acknowledgment of the primary role of the cecal microbiome in the pathogenesis of histomoniasis has provoked enormous interest in using specific nutritional interventions to strengthen host-defences (24). Unlike other microbiome-modulating strategies including phytogenics and prebiotics (which often suffer from rapid metabolic breakdown or require a pre-existing stable microbiota to function), probiotics provide a superior, active, and sustained biological shield. These live microorganisms have shown high potential as preventive agents, offering measurable health benefits to the host when taken in sufficient quantities (25, 26). By actively adding nutritional value to poultry feeds through supplementing certain desirable bacterial strains, producers can actively contribute to gastrointestinal tract integrity (27). These probiotic products have incredible potential to regulate the intestinal microbial community, strengthen epithelial barrier mechanisms, and promote strong mucosal immune responses (28, 29). As a result, a hardened gut environment will be much less favorable to the development of *H. meleagridis* and the consequent tissue invasion (22).

While the previous reviews have broadly covered poultry histomoniasis (5) or general probiotic applications in avian health, there is a lack of synthesized literature directly connecting probiotic mechanisms to the prevention of *H. meleagridis*. Therefore, the primary objective of this review is to bridge this gap. This manuscript specifically explains the various classes of probiotics available for poultry, the rigorous criteria for their selection, and their regulatory landscapes. Crucially, it evaluates the specific competitive interactions and immunomodulatory mechanisms through which probiotics inhibit *H. meeagridis* colonization, distinguishing this work from broader overviews.

## 2 Biology and pathogenesis of *Histomonas meleagridis*

The *Histomonas meleagridis* is an outstanding unicellular protozoan parasite which belongs to phylum Parabasalia (30). This very versatile pathogen has distinctive cellular features that have a drastic impact on its survival and virulence (31). The parasite has a pleomorphic character, i.e., the cellular structure of the parasite is actively adjusted to the location of the cellular parasites in the body of the living organism (32). When living in the luminal contents of the avian cecum, *Histomonas meleagridis* is mostly flagellated in form and can move in the liquid environment (33). But when the mucosal barrier is broken and the parasite invades the tissues beneath it, the parasite loses its single flagellum and becomes an amoeboid form (34). This important morphological change helps in tissue penetration and dissemination throughout the body (35). These virulence mechanisms rely heavily on the secretion of proteolytic enzymes and the active phagocytosis of the host cells, facilitating severe mucosal damage. Biologically, the parasitic organism is totally devoid of mitochondria and as such, its primary source of structuring essential cellular energy is using special intracellular organelles known as hydrogenosomes in a continuous anaerobic metabolism (36, 37).

**Figure 1:**
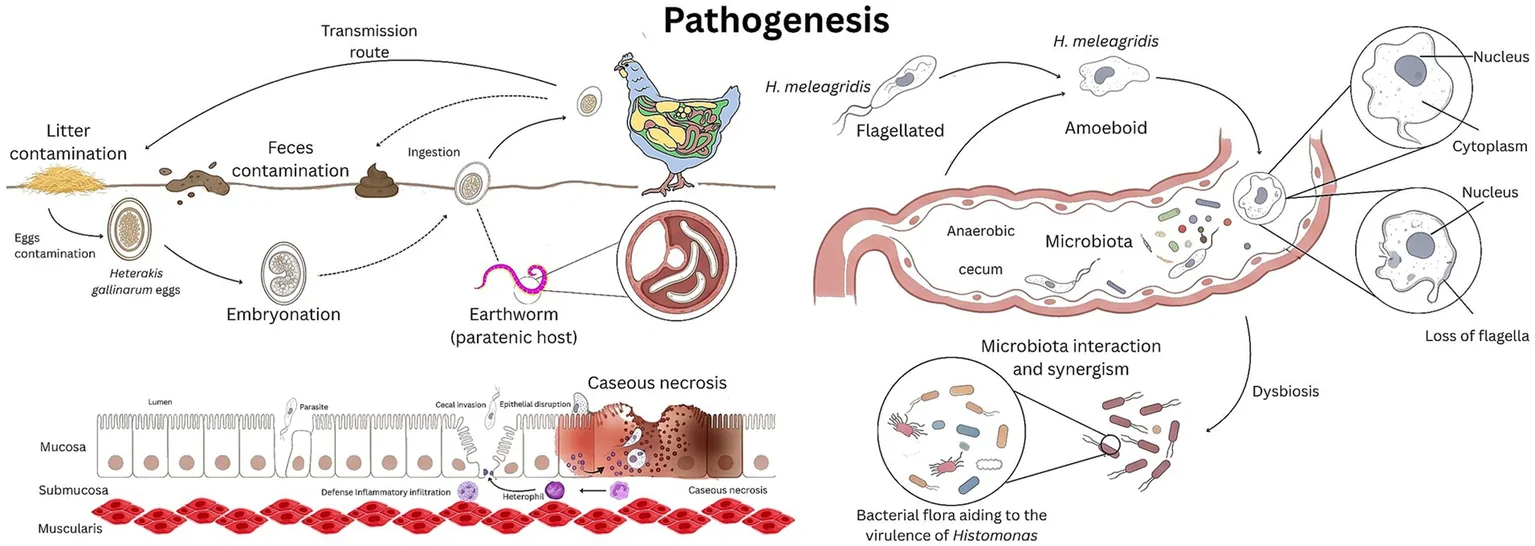
Pathogenesis and transmission cycle of *Histomonas meleagridis*. The schematic highlights oral ingestion of embryonated *Heterakis gallinarum* eggs, subsequent cecal colonization resulting in mucosal necrosis, and eventual systemic dissemination to the liver via portal circulation.

This pathogen has a complicated life cycle, which entails complex transmission mechanisms (38). The oral ingestion of embryonated eggs laid by the cecal nematode Heterakis gallinarum, which plays a central role in infecting birds, is the common way of infection (Figure 1) (39). The infected nematode eggs are often consumed by earthworms, which become paratenic hosts and create a gap in transmission between them and foraging poultry (40). Moreover, direct lateral transmission has been shown to be fast in commercial turkey flocks by a behavioral process called cloacal drinking, where contaminated feces are directly aspirated into the lower gastrointestinal tract by reverse peristalsis (6). The phenomenon justifies the tremendously fast spread of the disease in the case of an outbreak, even though the environmental frailty of the naked protozoan cells is low (41).

The pathogenesis of diseases starts as soon as the susceptible avian cecal lumen is successfully colonized by the parasites (42). Recent molecular studies indicate that this host–parasite interaction is directly mediated by the upregulation of specific inflammatory cytokines and the rapid disruption of the host tight junction proteins during initial colonization. The Histomonas proliferate rapidly and invade the fragile mucosal epithelium, causing a severe localized inflammatory response and widespread tissue necrosis (22). The result of this tremendous structural damage is thickened cecal walls and the development of a typical solid core of caseous with fibrin and necrotic cellular debris (43). After the initial enteric damage, the parasites commonly invade the local vascular network and directly enter the liver through the portal circulation (44). In the hepatic parenchyma, multifocal coagulative necrosis is severe because of a proliferation of *Histomonas meleagridis* (45). These characteristic pathological alterations give the liver a macroscopic appearance of circular depressions on its surface, which is often referred to as target like lesions (46). The systemic effects of such extreme cecal dysfunction coupled with severe hepatic failure inevitably result in extreme lethargy, rapid emaciation, characteristic sulfur colored diarrhea and ultimately fatal consequences in prone birds, resulting in severe annual economic losses for the global agricultural sector (47).

## 3 Avian cecal microbiome: composition, function, and dysbiosis

The gastrointestinal tract of birds is home to a truly remarkable and dynamic assembly of microscopic organisms, also known as the gut microbiome (48). Among the different parts of the digestive system, the ceca are paired parts and are the main location of microbial fermentation and host the greatest number of bacterial populations (49). The cecal microbiome of a healthy bird is largely dominated by bacteria of the phyla Firmicutes, bacteroidetes, Proteobacteria, and Actinobacteria (50). Among these high-level groups, Lactobacillus, Bifidobacterium, Clostridium, and Bacteroides are genera that are crucial to physiological functions (51). This complicated microbial ecosystem has enormous and unimaginable benefits to the host, as it helps to break down indigestible polysaccharides in the diet into nutrients that can be easily absorbed (52). The continuous production of short-chain fatty acids, mainly acetate, propionate, and butyrate, is one of the important functions that the commensal bacteria play (53). These critical metabolic byproducts act as the main source of energy in the epithelial cells covering the cecal wall, thus sustaining its strong structural integrity and proper mucosal functioning (54).

In addition to the nutritional benefits, the healthy cecal microbiome serves as an immunological organ which cannot be neglected and a physical barrier in the fight against pathogenic invasion (42). The occupying commensal organisms use the existing ecological niches and eat vital local nutrients, leaving the resources of the invading pathogens very scarce, an effect called competitive exclusion (55). Also, useful bacteria constantly discharge antimicrobial peptides and innately regulate the host’s innate immune system, maintaining a state of high immunological readiness without triggering detrimental inflammatory cascades (56). The finely tuned balance of this microbial network is hence not only pivotal in maintaining the overall health of the flock of poultry in agriculture but also optimum agricultural productivity (57).

But introduction of *Histomonas meleagridis* drastically disturbs this homeostatic condition and leads to a deep and harmful condition of microbial dysbiosis (22). The pathogenic process of the parasite actively changes the physical and chemical environment in the lumen of the cecum, making the environment extremely hostile to the useful anaerobic commensals (58). Experimental infection by microbial species has consistently shown a disastrous decrease in total microbial species richness and alpha diversity with the use of scientific analyses using advanced sequencing technologies (59). In particular, the protective populations of firmicutes and the beneficial Lactobacilli undergo a precipitous decrease, with some quantitative studies noting up to a 50% reduction in the relative abundance of these protective taxa, alongside a multifold increase in opportunistic proteobacteria (60). At the same time, such a devastating ecological change fills a vacuum in which opportunistic bacterial pathogens grow at a fast pace (61). *Escherichia coli* pathogenic strains that are usually maintained in the low levels in the normal and healthy avian stomach, proliferate and take over the altered cecal environment aggressively (62).

**Figure 2:**
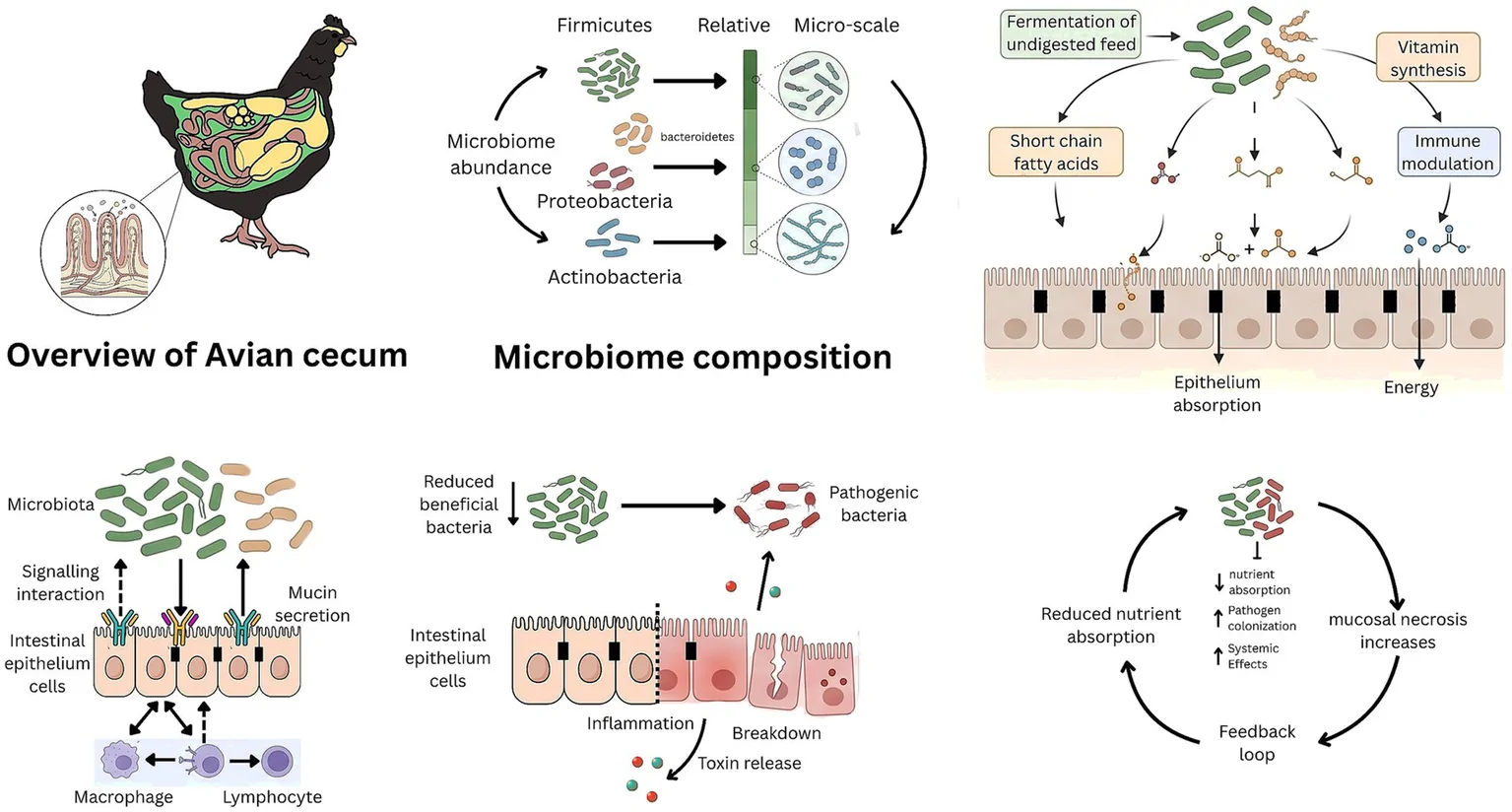
Homeostasis versus microbial dysbiosis in the avian cecum. The left panel illustrates a healthy microbiome dominated by beneficial Firmicutes producing protective short-chain fatty acids. The right panel demonstrates *H. meleagridis*-induced dysbiosis, characterized by a precipitous decline in commensal taxa and the proliferation of opportunistic *Proteobacteria*.

The resulting dysbiosis operates synergistically with the physical tissue damage caused by the protozoan parasite, forming a compounding loop of dire enteric destruction (63). A combination of the deep depletion of producers of beneficial short-chain fatty acids and a huge proliferation of proteobacteria, further impairs epithelial repair processes and the explosive inflammatory cascade (Figure 2) (64). The researchers have indeed determined that the widespread increase in the number of secondary bacterial invaders plays a major role in aggravating the major lesions of histomoniasis (65). However, it is worth noting that conflicting findings exist regarding the exact succession of this dysbiosis; while some studies suggest microbial shifts precede severe clinical lesions, others argue that dysbiosis is strictly a secondary consequence of the parasite-induced mucosal necrosis. Moreover, the impaired mucosal barrier permits the bacteria to infiltrate deeper tissues and get into the systemic circulation, which in many cases results in fatal secondary colibacillosis (66). This complex interaction can be conclusively understood to explain why regaining normal cecal microbial homeostasis is a top priority in creating effective disease reduction strategies to be applied to vulnerable commercial poultry flocks that work across the globe (57).

## 4 Probiotics: classes, selection criteria, and regulatory landscape

Probiotics represent an innovative paradigm in poultry disease management, defined as live microbes that have a positive health effect on the host when used in optimal amounts (67). In commercial agriculture, the wide range of useful probiotic organisms typically can be categorized into three main taxonomic groups (68). The most popular group is lactic acid bacteria, which include, conspicuously, some of the well-known species of the genera Lactobacillus, Enterococcus, Pediococcus and Bifidobacterium (69). These selected non-pathogenic bacteria are greatly appreciated because of their remarkable capacity to reduce the local intestinal potential of hydrogen very rapidly by the abundant production of organic acids (70). The second large functional group comprises the spore-forming bacteria, which are mostly of the hardy Bacillus type, as in *Bacillus subtilis* and *Bacillus licheniformis* (Table 1) (71). These extraordinary spore formers have the advantage of a unique manufacturing process because of their very high resistance to the high levels of physical heat produced in the commercial feed pelleting process (72). The third category includes useful fungi, mainly the strong yeast *Saccharomyces cerevisiae*, which has specific enzyme functions facilitating digestion and the unique ability of binding mycotoxins that are harmful and are found in contaminated feedstuffs (73). Recently, next generation probiotics, such as specific commensal strains of *Akkermansia* or genetically engineered microbial consortia, have also emerged as highly targeted future options for the precision microbiome modulation.

**Table 1:** Probiotic-based modulation of the cecal microbiome in poultry and its relevance to *Histomonas meleagridis* control.

Sr. No.	Probiotic Type	Representative Species/Strains	Primary Site of Action	Key Mechanisms of Microbiome Modulation	Effects on Cecal Microbiota Composition	Impact on Host Immunity	Evidence from Poultry Studies	Results	Limitations	References
1	Lactic Acid Bacteria (LAB)	*Lactobacillus acidophilus*, *L. salivarius*	Cecum, small intestine	Organic acid production, bacteriocins, competitive exclusion	Increased Firmicutes, reduced Enterobacteriaceae	Increased IgA, cytokine modulation	Improved gut health and pathogen reduction in broilers	Reduces bacterial support required for parasite survival	Strain-specific variability	(118)
2	Bifidobacteria	*Bifidobacterium bifidum*, *B. animalis*	Cecum	SCFA production, fermentation modulation	Increased beneficial anaerobes	Anti-inflammatory, barrier strengthening	Improved microbial balance in poultry	Alters metabolic environment unfavorable to parasite	Low persistence in poultry gut	(119)
3	Spore-forming bacteria	*Bacillus subtilis*, *B. licheniformis*	Entire gut including cecum	Enzyme production, antimicrobial peptides	Increased diversity, reduced pathogens	Enhanced innate immunity	Improved growth and pathogen resistance	Reduces microbial partners aiding parasite	Dose-dependent response	(120)
4	Yeast probiotics	*Saccharomyces cerevisiae*	Cecum	Toxin binding, microbiome modulation	Increased SCFA-producing bacteria	Enhanced antibody response	Improved gut health and stress resistance	Stabilizes microbiome indirectly limiting parasite	Less direct antimicrobial activity	(121)
5	Multi-strain probiotics	LAB + *Bacillus* + *Bifidobacterium*	Whole gut	Synergistic microbiome modulation	Increased diversity and stability	Broad immune enhancement	Superior to single strains in poultry	Comprehensive disruption of parasite-supportive microbiota	Formulation complexity	(122)
6	Synbiotics	Probiotics + FOS/MOS	Cecum	Enhanced colonization, fermentation stimulation	Increased beneficial bacteria, SCFA production	Improved immune responses	Enhanced gut health and disease resistance	Creates an unfavorable environment for parasite persistence	Requires optimization	(123)
7	Enterococcus spp.	*Enterococcus faecium*	Cecum	Bacteriocin production, competitive exclusion	Stabilized microbiota	Immune stimulation	Reduced enteric pathogens in poultry	Limits bacterial synergy with parasites	Safety concerns in some strains	(124)
8	Butyrate-producing bacteria	*Clostridium butyricum*	Cecum	Butyrate production, anaerobic modulation	Increased SCFA producers	Anti-inflammatory, epithelial protection	Improved gut integrity	Butyrate may inhibit parasite colonization	Sensitive to environmental conditions	(125)
9	Direct-fed microbial consortia	Mixed microbial cultures	Cecum	Ecological niche occupation, microbiome restoration	Rapid normalization of dysbiosis	Enhanced immune readiness	Used in competitive exclusion models	Prevents establishment of parasite-supporting microbiota	Variability in composition	(126)
10	Postbiotics	SCFAs, bacteriocins	Cecum	pH modulation, antimicrobial metabolites	Supports beneficial microbiota indirectly	Anti-inflammatory effects	Emerging applications in poultry	Alters environment reducing parasite survival	Limited direct histomonosis data	(127)
11	Microbiome interaction studies	Bacteria supporting *H. meleagridis*	Cecum	Symbiotic interaction with parasite	Increased Proteobacteria during infection	Dysregulated immune response	Demonstrated bacterial dependence of parasites	Direct evidence of microbiome role in disease	Not probiotic but critical context	(5)

The effective screening followed by the commercialization of an effective poultry probiotic entails careful screening against a stringent set of biological selection criteria (74). To begin with, the candidate microbial strain should exhibit total non-pathogenicity and total safety for the target avian host and the end-user human consumer (75). Crucially, probiotic efficacy is highly strain-specific and dose-dependent; a formulation that successfully provides immunomodulation in one specific *Lactobacillus* strain may be completely ineffective in a closely related strain, necessitating precise dosage optimization. Furthermore, critical limitations must be evaluated, including the risk of variable *in vivo* efficacy due to flock genetics, transient colonization requiring continuous supplementation, and the potential risk of transferring antimicrobial resistance genes. After the preliminary safety tests, the microorganism should have extreme physiological stability (76). In particular, the probiotic cells have to be able to endure the remarkably hostile environmental conditions, they must face in the upper gastrointestinal tract, which must exhibit a high level of tolerance to strong gastric acids and concentrated bile salts (77). Moreover, a good probiotic strain should have the special biological ability to absorb the host intestinal mucosal epithelium (78). This important adhesive property assures stable long-term colonization and proactively averts the beneficial bacteria, which are quickly washed out in the normal peristaltic motions (79). Also, it is preferable that the chosen strains possess a high rate of reproductive growth and be actively capable of producing natural antimicrobial compounds, called bacteriocins, to maximize their ability to competitively exclude invading pathogens (80).

In addition to these rigorous biological conditions, the regulatory situation in the commercial production of poultry in the global context with the use of probiotics is extremely complicated and geographically diverse (81). In the United States, probiotic feed additives have to pass intense scrutiny performed by the Food and Drug Administration before being allowed to attain the much desired recognized as safe status before commercial sale (82). This is a complicated regulatory procedure that requires extensive scientific evidence of the safety and the regular effectiveness of the bacterial strain (83). Conversely, the European Union enforces a highly stringent regulatory framework monitored by the European Food Safety Authority (84). The probiotics are officially considered zootechnical feed additives under European legislation (85). Manufacturers should provide large volumes of empirical dossiers to unequivocally show the not only safety of the microbial product, but its quantifiable effectiveness in enhancing the performance of animals or to certain environmental effects (86). These significant regional regulatory disparities have been and continue to be an absolute necessity in understanding and maneuvering global agricultural suppliers in the effort to adopt one of the most innovative microbiomes targeted biological tools to counter the devastating persistent threat of histomoniasis (87).

## 5 Mechanisms of probiotics against *Histomonas meleagridis*

**Figure 3:**
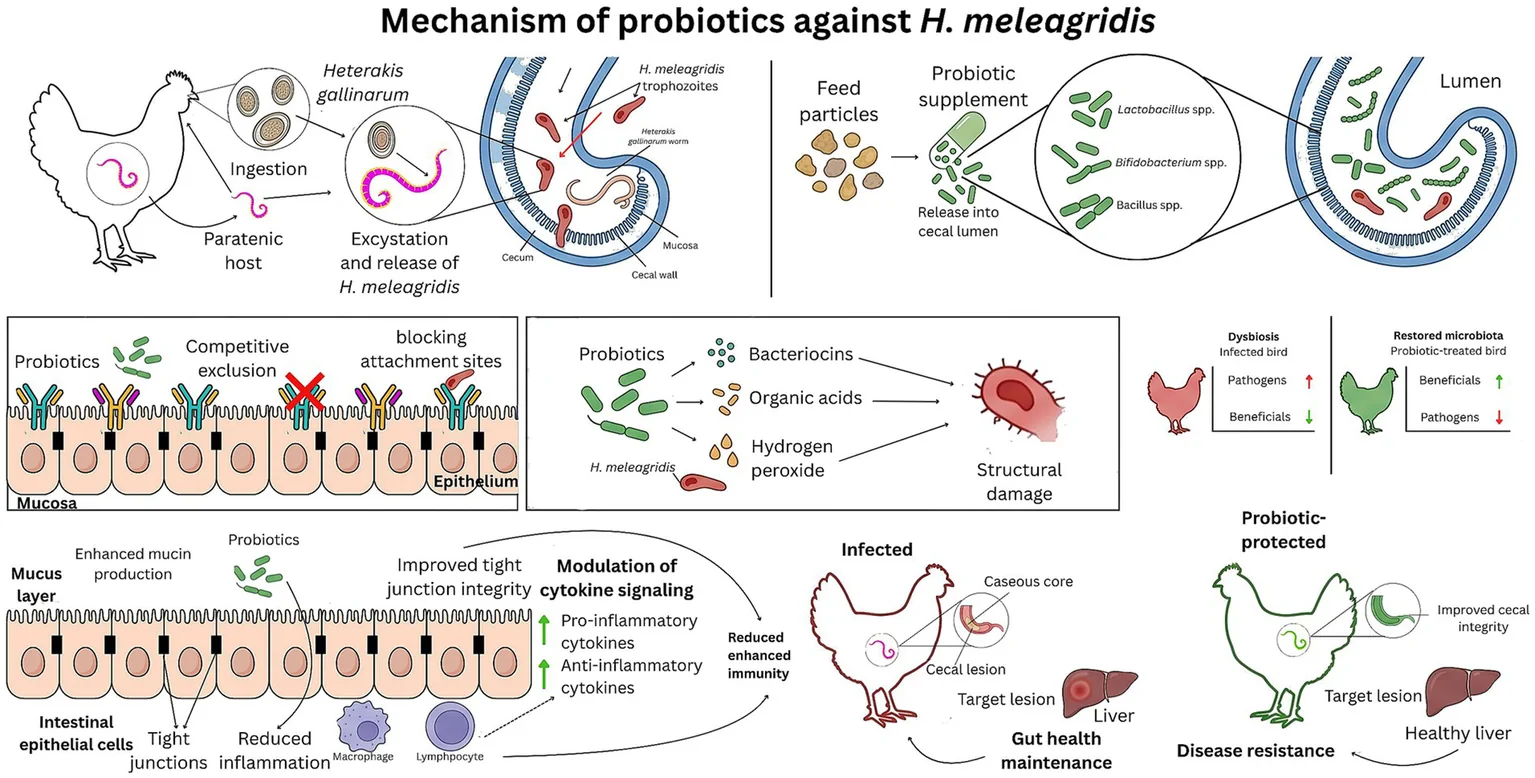
Proposed molecular and ecological mechanisms of probiotics against *H. meleagridis*. Key actions include competitive exclusion at the mucosal interface, environmental acidification via SCFA production, fortification of tight junction proteins, and targeted immunomodulation via Toll-like receptor (TLR) signaling.

Probiotics use multi-faceted biological processes to regulate the avian cecal microbiome and to actively defend against infection with *Histomonas meleagridis* (22). The competitive exclusion principle is very fundamental to the main defence mechanism that is used by these useful microorganisms (88). By prophylaxis, large populations of probiotic bacteria quickly colonize the cecal mucosa, effectively covering all the sites available in terms of physical attachment to the epithelial lining (89). These protective strains form a strong structural barrier establishing a formidable structural barrier that effectively blocks the parasite’s flagella from adhering to and penetrating the susceptible cecal wall (Table 2) (22). At the same time the growing, fast-growing probiotic organisms are in intense competition with the invading protozoan parasite over the limited available essential nutrients in the local microenvironment, virtually starving the pathogen and severely restricting its ability to undergo massive exponential growth (Figure 3) (90).

**Table 2:** Role of probiotics in poultry gut health and their translational relevance to *Histomonas meleagridis* control.

S. No.	Study type	Animal model	Probiotic intervention	Dosage & duration	Target outcome measured	Observed microbiome changes	Disease/pathogen model	Key findings	Translational relevance to *H. meleagridis*	Limitations	References
1	*In vivo*	Broiler chickens	*Lactobacillus salivarius*	10^7^–10^8^ CFU/day for 35 days	Growth performance, gut health	Increased Firmicutes, reduced *E. coli*	*Salmonella enterica* challenge	Reduced pathogen colonization and improved gut integrity	Suggests reduction of bacteria supporting parasite growth	No direct protozoan model	(120)
2	*In vivo*	Broiler chickens	*Bacillus subtilis*	10^6^ CFU/g feed for 42 days	Feed conversion, immunity	Increased microbial diversity	*Clostridium perfringens*	Reduced necrotic enteritis severity	Demonstrates microbiome stabilization relevant to histomonosis	Indirect evidence	(128)
3	*In vivo*	Broiler chickens	Multi-strain probiotic	Mixed strains for 28 days	Immune markers, microbiota	Increased Lactobacillus spp.	No pathogen challenge	Enhanced immune response and gut stability	Supports resistance to microbial dysbiosis	No infection model	(118)
4	*In vivo*	Turkeys	*Enterococcus faecium*	10^8^ CFU/day for 21 days	Immune response, pathogen load	Reduced Enterobacteriaceae	*E. coli* infection	Lower pathogen load and improved immunity	Limits bacterial synergy required by parasite	Species-specific variability	(124)
5	*In vivo*	Broiler chickens	*Saccharomyces cerevisiae*	0.5–1 g/kg feed for 35 days	Gut morphology, immunity	Increased SCFA producers	Heat stress model	Improved gut barrier and immune resilience	Indirectly limits parasite invasion	No pathogen-specific model	(121)
6	*In vivo*	Broiler chickens	Synbiotic (LAB + MOS)	1 g/kg feed for 28 days	Microbiome composition, immunity	Increased beneficial anaerobes	*Salmonella* challenge	Reduced pathogen colonization	Enhances resistance to microbiome disruption	Requires optimized combinations	(123)
7	*In vivo*	Broiler chickens	*Clostridium butyricum*	10^7^ CFU/g feed for 42 days	SCFA levels, gut integrity	Increased butyrate production	*E. coli* challenge	Improved epithelial integrity and reduced inflammation	Butyrate environment may inhibit parasite	Limited parasite-specific data	(125)
8	*In vivo*	Cecal culture model	LAB strains	Variable	Pathogen inhibition	Reduced pathogenic bacteria	*E. coli*, *Salmonella*	Direct inhibition of pathogens	Suggests removal of parasite-supporting bacteria	In vitro limitations	(129)
9	*In vivo*	Chickens	Direct-fed microbial mix	Commercial dose for 35 days	Performance, microbiome	Rapid microbiota normalization	No challenge	Improved gut stability	Prevents dysbiosis required for parasite establishment	Lack of pathogen model	(126)
10	*In vivo*	Turkeys	Microbiome interaction study	Not applicable	Cecal microbiota changes	Increased Proteobacteria during infection	*H. meleagridis*	Demonstrated bacterial dependence of parasite	Direct evidence supporting probiotic strategy	Not an intervention study	(5)
11	*In vivo*	Broiler chickens	Multi-strain probiotic	10^8^ CFU/day for 42 days	Immune gene expression	Increased Lactobacillus spp.	Coccidiosis model	Reduced lesion scores and improved immunity	Indicates applicability to protozoan infections	Different protozoan species	(130)
12	*In vivo*	Broiler chickens	*Bacillus licheniformis*	10^6^ CFU/g feed for 35 days	Growth and microbiota	Reduced pathogenic bacteria	No challenge	Improved feed efficiency and gut health	Supports microbiome stability against parasite	Indirect evidence	(131)

Probiotics actively manipulate the chemical environment of the gastrointestinal tract to form an extremely hostile environment in which pathogens cannot survive (91). Bacteria that produce lactic acid, especially, can produce abundant levels of organic acid that are directly released into the cecal lumen (92). The short-chain fatty acids (SCFAs) are important signaling molecules that bind to the host G-protein-coupled receptors (GPCRs) GPR41 and GPR43 on the intestinal epithelium. This pathway of metabolite sensing acts directly as an inhibitor of NF-κB activation and also blocks an unchecked inflammatory cascade usually activated by parasite. Moreover, the production of very specific bacteriocins like enterocins and plantaricins directly compromises the integrity of the membrane of synergistic pathobionts, thus fundamentally altering the microbial support network that is indispensable for the survival of *H. meleagridis* (93). *Histomonas meleagridis* is highly sensitive to acidic environments, and this artificially induced luminal acidification has a drastic motility inhibitory effect, as well as a growth- and viability-limiting effect on the protozoan cells (5). Additionally, some probiotic strains produce and release strong antimicrobial peptides (often referred to as bacteriocins), which are produced naturally (94). Although mainly acting on opportunistic secondary bacterial invaders, such as *Escherichia coli*, the repression of these synergistic bacterial pathogens indirectly debilitates the virulence factors (57). Which are extremely dependent on these particular bacteria to maintain their own metabolic activity and cause severe disease. It must be clearly distinguished, however, that while environmental acidification and bacteriocin production are experimentally demonstrated mechanisms against secondary bacterial invaders, their direct *in vivo* lethal mechanisms against *H. meleagridis* specifically remain largely hypothetical. Currently, these direct anti-protozoal mechanisms are extrapolated from models of other avian diseases.

Importantly, probiotics have an active role in strengthening the structural integrity of the intestinal barrier of the host (95). In a massive outbreak of histomoniasis, the parasite attacks the mucosal epithelium with great severity, to give it an opportunity to migrate systemically to the liver (22). This devastating mechanism is directly opposed by beneficial probiotic bacteria, which activate the expression of essential tight junction proteins, namely, claudins and occludins, between the epithelial cells to each other (96). Such strengthening of the structure greatly limits the capacity of the protozoan to penetrate the intestinal wall, thus avoiding disastrous secondary liver death (63).

The immunomodulatory properties of probiotics provide profound benefits to the avian innate defense system (97). Probiotic organisms actively interact with host pattern recognition receptors, particularly Toll-like receptors (TLRs), on gut-associated lymphoid tissue. This TLR signaling cascade modulates specific cytokine profiles, often downregulating pro-inflammatory markers while upregulating protective cytokines, which subsequently induces increased local secretory immunoglobulin A production (98). These specialized protective antibodies are free to circulate in the layer of intestinal mucus and, in effect, inactivate the parasite before it can initiate massive tissue damage (99). Certain beneficial strains can carefully control the systemic host inflammatory response (100). Probiotics prevent the extreme collateral tissue injury that is normally seen with severe histomoniasis by actively modulating the local cytokine response towards an inappropriate, unchecked state of destructive inflammation (101). This complex interaction of competitive exclusion, environmental acidification, structural reinforcement and active immune stimulation (102). Therefore, gives probiotics a very comprehensive and biologically sustainable prophylactic defence against this devastating avian pathogen, ensuring agricultural production systems around the world to future generations of humans (67).

### 5.1 Direct vs. indirect evidence of efficacy

One of the major limitations in the present histomoniasis studies is the reliance on indirect evidence. Most evidence for probiotic effectiveness (e.g., competitive exclusion and mucosal fortification) is from *in vivo* poultry study data with other enteric pathogens, such as *Salmonella enterica* or *Escherichia coli*. Although these studies demonstrate the ability of probiotics to reduce microbial dysbiosis, there are very few studies with direct in vivo experimental data using specific challenge models with *H. meleagridis*. Empirical validation in the future should carefully distinguish between general enteric health from direct anti-protozoal activity of probiotics to establish their clinical effectiveness against histomoniasis.

## 6 Comparative analysis with other prophylactic strategies

In the assessment of overall disease mitigation measures in commercial poultry, a thorough comparative study of the use of probiotics versus other possible prophylaxis is critical (103). The most noticeable alternative interventions are currently the extensive use of phytogenic feed supplements, the strategic use of specific anthelmintic drugs, and the continued experimentalizing of commercial vaccines (104). Phytogenic compounds are mostly extracts of plants of high concentration and essential oils of high volatility that are produced by plants such as oregano and garlic and have strong direct antimicrobial and antiprotozoal effects (105). In contrast to living probiotic organisms, which actively reproduce and keep colonizing the intestinal tract to maintain a sustained biological shield, phytogenic compounds are functionally more like natural chemical therapies (Table 3) (106). Although these potent compounds of plant origin can quickly inhibit the growth of histomonas *in vitro*, in vivo their effectiveness is often inconsistent because of irregular absorption and rapid metabolic breakdown in the avian intestinal tract (107). Probiotics thus always have a much greater benefit in terms of intestinal long-term survival and permanent mucosal defence (108). The other historically prevalent prophylactic measure is the regular use of anthelmintic drugs, e.g., fenbendazole, to exclude a cecal nematode *Heterakis gallinarum* (109). Because this specific nematode acts as the primary biological vector for *H. meleagridis*, eliminating the worm theoretically prevents the establishment of the lethal protozoa (40). But there is a drastically growing crisis in the modern agricultural industry in terms of extensive anthelmintic resistance (110). Rampant use of these chemical dewormers has quickly adapted to highly resistant nematode strains, making this conventional prophylactic route less reliable (111). On the other hand, the probiotics do not serve to attack the nematode carrier but actively enhance the host environment regarding the final protozoan invader without any reference to the critical evolutionary traps in the development of the chemical resistance (112). Lastly, although live attenuated vaccines are a very promising prophylaxis option, significant technical hurdles remain the primary obstacle to the commercialization of live attenuated vaccines (113). The existing experimental histomoniasis vaccines are so delicate that they need highly complicated, individualized routes of administration and are thus cost-prohibitive in a large-scale commercial turkey enterprise of thousands of birds (22). Very contrastingly, with the spore forming probiotics, we have an extremely stable, highly scalable, and easily administered solution using standard automated feed delivery systems (114). From a practical standpoint, this ease of implementation ensures high cost-effectiveness under commercial poultry conditions, drastically reducing the labor costs associated with individualized administration. A major limitation, however, is that non-spore-forming live probiotics are highly sensitive to environmental fluctuations; they require meticulous temperature and humidity control during storage, transport, and field application to maintain optimal cellular viability. This presents a significant logistical and financial hurdle in commercial settings lacking robust cold-chain infrastructure (115). They nevertheless are the most cost-effective, ecologically friendly, and extremely biologically efficient prophylaxis measure in the poultry industry today (116). Which is actively fighting the devastating effects of frequent histomoniasis outbreaks affecting all parts of the agricultural industry on a global scale (117).

**Table 3:** Comparative analysis of prophylactic strategies.

Prophylactic strategy	Mechanism of action	Primary advantages	Disadvantages & limitations	Cost-effectiveness & implementation
Probiotics	Competitive exclusion, immunomodulation, and mucosal barrier fortification.	Actively reproduce for sustained biological shield; ecologically friendly; does not induce resistance.	Live non-spore-forming strains are sensitive to environmental fluctuations; requires cold-chain storage.	Highly cost-effective; easily administered at scale using standard automated feed delivery systems.
Phytogenics (e.g., Oregano, Garlic extracts)	Direct antimicrobial and antiprotozoal action via concentrated plant extracts and essential oils.	Natural origin; demonstrates rapid *in vitro* inhibition of *H. meleagridis*.	Inconsistent *in vivo* efficacy due to irregular absorption and rapid metabolic breakdown in the gut.	Moderate; variable efficacy and lack of sustained mucosal defense can reduce overall return on investment.
Anthelmintics (e.g., Fenbendazole)	Eradication of the cecal nematode *Heterakis gallinarum*, the biological carrier of the protozoan.	Historically proven to break the transmission cycle and prevent protozoan establishment.	Rampant and growing anthelmintic resistance in nematode strains; offers no direct defense against the protozoan itself.	Decreasing cost-effectiveness due to high failure rates and adaptation of resistant nematode strains.
Live attenuated vaccines	Stimulation of adaptive immune memory via experimental attenuated strains.	Offers highly specific, acquired systemic immunity against the pathogen.	Extremely delicate; technically challenging to commercialize and stabilize.	Cost-prohibitive for large-scale commercial flocks; requires highly complicated, individualized administration routes.

## 7 Host-specific responses: turkeys vs. chickens

A crucial factor in probiotic application is the stark biological divergence in disease susceptibility between avian hosts. *H. meleagridis* is very virulent and can cause mortality of up to 100% in turkeys, with rapid necrosis of the liver. On the other hand, chickens have usually less severe clinical signs and are considered to be asymptomatic reservoirs. Such difference is closely connected with variations in their baseline cecal microbiomes and innate mucosal immunity. Thus, the strain that works to prevent dysbiosis in the chicken model may not be able to colonize or modulate in the highly dynamic, susceptible gut system of the turkey. To bridge this translational gap a species-specific probiotic formulation which meets the unique physiological requirements of turkeys is required.

## 8 Future perspectives

The future of prevention of commercial histomoniasis depends on the fast development of precision microbiome engineering technologies. The vast therapeutic potential of multi-strain probiotic combinations is already under investigation by researchers in terms of specifically developed mixtures of probiotics, which are proposed to synergize the effects of competitive exclusion and immunomodulation. Careful mixing of particular lactic acid bacteria with strong spore formers could lead to scientists developing very strong and well-rounded protective physiological barriers in the avian gut. Moreover, recent advances in new genetic sequencing can be used to specifically identify completely new commensal bacterial species specially adapted to adverse cecal conditions. It is very likely that future research will concentrate on the use of more sophisticated bioengineering methods that can be used to directly improve the natural bacteriocin production potential of these native isolates. With regulatory structures invariably evolving to fit these advanced biologic advances, next generation precision probiotics will revolutionize commercial agriculture disease management, providing highly targeted, environmentally friendly, and highly effective prophylaxis against this parasite. To bridge the current knowledge gaps, future research must prioritize moving beyond *E. coli* or chicken models to establish reproducible *H. meleagridis* challenge models specifically in tukerys, allowing for direct quantification of anti-protozoa probiotic efficacy. However, utilizing transcriptomics and metabolomics to track how specific probiotics strains dramatically interact with cecal mucosa during an active protozoan infection. Prioritizing the isolation and bioengineering of spore-forming commensals that can survive commercial feed pelleting and mitigate the logistical cold-chain limitations of current vegetative strains.

## 9 Conclusion

The global resurgence of *Histomonas meleagridis* following the ban on traditional pharmacological treatments represents a critical crisis in contemporary poultry production. The modulation of the avian cecal microbiome by targeted probiotics represents a promising biological approach to counteract dysbiosis caused by pathogens, however, there is a critical need to recognize that the current amount of evidence is predominantly based on indirect extrapolation from the bacterial enteric model. Although few data are available for *in vivo* challenge with protozoa, the mechanisms by which probiotics exert their effects (competitive exclusion, luminal acidification, epithelial fortification, and selective TLR-mediated immunomodulation) suggest that they have tremendous protective potential for enteric pathogen establishment. Although major logistical issues, such as strict strain selection, product stability, and extremely variable regional regulatory compliance continue to be challenges in the global context, the biological benefits of microbiome targeted therapies are undeniable, and the logistical challenges they present are not as significant as they might seem. Finally, the complete implementation of these sophisticated biological interventions in the complex flock management procedures will be completely necessary for ensuring sustainable production in agriculture. This entirely ecologically sustainable paradigm shift is the most viable long-term direction in ensuring overall world food security, and at the same time addresses the most essential animal welfare needs as well as the most important human consumer safety needs of today.
